# B chromosome in the beetle *Coprophanaeus cyanescens* (Scarabaeidae): emphasis in the organization of repetitive DNA sequences

**DOI:** 10.1186/1471-2156-13-96

**Published:** 2012-11-06

**Authors:** Sarah Gomes de Oliveira, Rita Cassia de Moura, Cesar Martins

**Affiliations:** 1 Department of Morphology, Bioscience Institute, UNESP - Sao Paulo State University, Botucatu, SP, 18618-970, Brazil; 2 Department of Biology, Biological Sciences Institute, UPE - Pernambuco State University, Recife, PE, 50100-130, Brazil

**Keywords:** Chromosomal rearrangements, Heterochromatin, Multigene families, Supernumerary chromosome, Transposable elements

## Abstract

**Background:**

To contribute to the knowledge of coleopteran cytogenetics, especially with respect to the genomic content of B chromosomes, we analyzed the composition and organization of repetitive DNA sequences in the *Coprophanaeus cyanescens* karyotype. We used conventional staining and the application of fluorescence *in situ* hybridization (FISH) mapping using as probes *C*_*0*_*t*-1 DNA fraction, the 18S and 5S rRNA genes, and the *LOA-like* non-LTR transposable element (TE).

**Results:**

The conventional analysis detected 3 individuals (among 50 analyzed) carrying one small metacentric and mitotically unstable B chromosome. The FISH analysis revealed a pericentromeric block of *C*_*0*_*t*-1 DNA in the B chromosome but no 18S or 5S rDNA clusters in this extra element. Using the *LOA-like* TE probe, the FISH analysis revealed large pericentromeric blocks in eight autosomal bivalents and in the B chromosome, and a pericentromeric block extending to the short arm in one autosomal pair. No positive hybridization signal was observed for the *LOA-like* element in the sex chromosomes.

**Conclusions:**

The results indicate that the origin of the B chromosome is associated with the autosomal elements, as demonstrated by the hybridization with *C*_*0*_*t*-1 DNA and the *LOA-like* TE. The present study is the first report on the cytogenetic mapping of a TE in coleopteran chromosomes. These TEs could have been involved in the origin and evolution of the B chromosome in *C. cyanescens*.

## Background

Eukaryote genomes are composed of classical genes and genetic elements, including transposable elements (TEs), B chromosomes and several cytoplasmic factors that do not follow Mendelian laws of inheritance [1]. B chromosomes (also called supernumerary or accessory chromosomes) are not essential for the life of a species and are thus considered “dispensable” additional chromosomes. B chromosomes have been observed in approximately 15% of living species [1-4]. Most B chromosomes are heterochromatic and composed of repetitive DNA sequences, supporting the idea that these chromosomes are non-coding. However, some B chromosomes show the presence of active genes [5-7]. B chromosomes demonstrate an irregular behavior during mitosis and meiosis that allows them to accumulate in the germ line in a non-Mendelian pattern of inheritance [3,8]. Although B chromosomes have been the focus of intensive work in a diversity of eukaryotic species [9-17], several questions concerning their origin, evolutionary mechanism and function remain unanswered.

In Coleoptera, the presence of B chromosomes has been described in approximately 80 species belonging to several families, including Buprestidae [18], Cantharidae [19], Cicindelidae [20] and Scarabaeidae [21,22]. In general, the studies in Coleoptera have concentrated on the presence or absence of B chromosomes in species, with few reports covering their frequency in populations and/or their molecular content [18,21-23]. There are a few reports on the presence of B chromosomes in the Scarabaeidae family, including species of the Scarabaeinae and Cetoniinae subfamilies [21,22]. Among scarabaeines, the *Coprophanaeus* species (Phanaeine) showed similar karyotypes consisting of 2n = 20 and meta-submetacentric chromosomes with a gradual reduction in size, three types of sex chromosomes mechanisms (XY, Xy, XY_p_), a high amount of constitutive heterochromatin, and there is no description of B chromosomes for this group until now [24-26]. Besides their karyotype characteristics, the phaneines are restricted to the Neotropical region and play an important role in the ecosystems including nutrient recycling [27-29].

Although the cytogenetic mapping of repetitive DNA sequences has been performed for several species of coleopterans, the data are limited to the analysis of satellite DNA, rRNA and H3 histone genes e.g. [22,24-26,30-34]. Based in the heterochromatic nature of the B chromosomes and that several families of TEs are particularly enriched in heterochromatin, it is particularly interesting the analysis of TE sequences in relation to their organization in B chromosomes. Considering the gap of knowledge on the genomic content of Coleoptera B chromosomes, the present work performed molecular cytogenetic mapping of repetitive DNAs in the beetle *Coprophanaeus cyanescens*, with emphasis in the investigation of the B chromosome.

## Results

**Figure 1:**
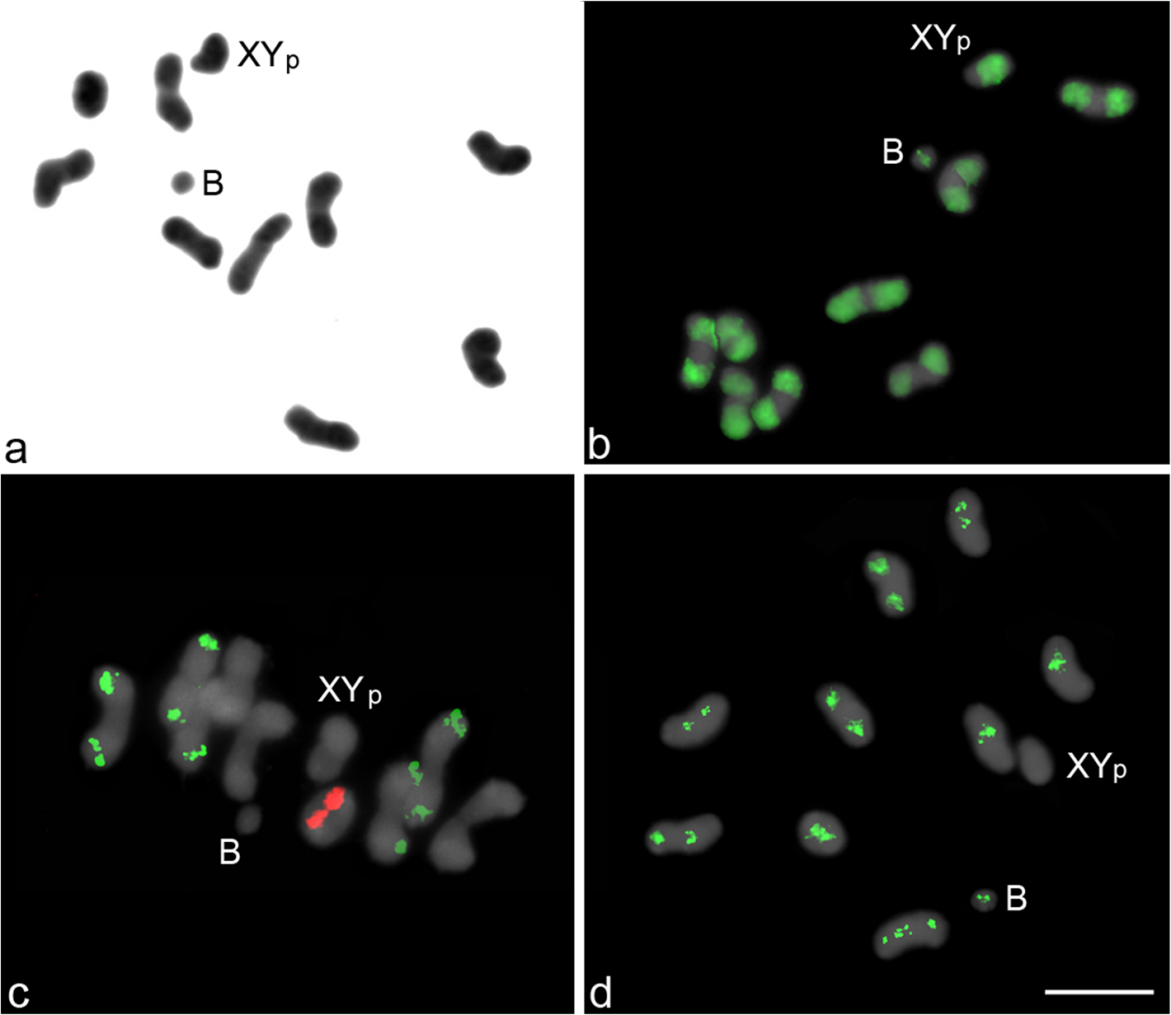
**Metaphase I stages of *Coprophanaeus cyanescens *carrying 1 B chromosome.** Conventional staining (**a**), FISH mapping of *C*_*0*_*t*-1 DNA (**b**), 18S (green) and 5S (red) rRNA genes (**c**) and *LOA-like* non-LTR retrotransposon (**d**). The B chromosome and the XY_P_ sex chromosomes are indicated. Bar = 5 μm

The standard karyotype observed in *C. cyanescens* was 2n = 20, XY_p_ (“p” refers to a “parachute” meiotic conformation between the X and Y), with meta-submetacentric chromosomes that showed a gradual reduction in size (Figure 1a). In addition, three individuals among the 50 analyzed (6%) carried 1 small-sized B meta-submetacentric chromosome. For each individual carrying the B chromosome, at least 30 metaphase I stages were analyzed, and 13.8% of the cells did not present the extra chromosome, indicating mitotic instability. The B chromosome had a condensation pattern similar to that of the autosomal chromosomes and was easily recognized as a small univalent structure in metaphase I (Figure 1).

The FISH analysis using the *C*_*0*_*t*-1 DNA probe revealed positive hybridization in the long arms of all the autosomal chromosomes and the X and Y chromosome and in a pericentromeric block in the B chromosome (Figure 1b). The chromosomal mapping using the 18S and 5S rDNA probes showed clusters on distinct chromosomes (Figure 1c). The 18S rDNA clusters were observed at nine sites (four autosomal pairs plus one single chromosome), and the 5S rDNA clusters were observed at two sites (one autosomal pair) (Figure 1c). None of the rDNA probes hybridized with the B chromosome (Figure 1c).

Analysis of the non-LTR retrotransposon sequence (hereafter named the *LOA-like* non-LTR retrotransposon), which was isolated by polymerase chain reaction (PCR) and subsequently cloned, revealed a segment of 223 bp that shared ~65% similarity to the *Baggins-1_Nvi* family previously identified in *Nasonia vitripennis*[35]. The alignment of these sequences is shown in Additional file 1. FISH analysis using probes for the *LOA-like* element revealed large pericentromeric blocks in eight autosomal bivalents and the B chromosome and a pericentromeric block extending to the short arm in one autosomal pair; a positive hybridization signal was not observed in the sex chromosomes (Figure 1d).

## Discussion

### Basic characteristics of the *C. cyanescens* karyotype

The basic karyotype structure for *C. cyanescens* (composed of 2n = 20, XY_p_, with meta-submetacentric chromosomes) is in concordance with previous karyotype data reported for *Coprophanaeus* species [26,31,32]. However, this is the first study to identify a B chromosome in this species as well as in the Phanaeini tribe. In contrast to the small size of the B chromosome observed in *C. cyanescens*, the B chromosomes were medium- or large-sized in the other Scarabaeidae species [21,22,36]. In *Onthophagus vacca*, the presence of one medium-sized B chromosome was observed with the presence of heterochromatin in its centromeric region, whereas *Onthophagus similis* and *O. gazella* showed respectively medium- and small-sized B chromosomes; however, there was no information about the heterochromatic pattern. Large heterochromatic B chromosomes, ranging in number from three to nine, were detected in all the specimens studied for *Bubas bubalus*[21]. Individuals carrying one heterochromatic B chromosome in two populations of *Dichotomius geminatus*, corresponding to an average prevalence rate of 20.93% and 25.00% in each of the populations, were observed [22].

The frequency with which B chromosomes are detected in natural populations varies widely between populations. B chromosomes can be present in high frequencies based on the degree to which a species can tolerate the extra chromosome and their power of accumulation [23]. It is difficult to determine the factors that are involved in the low frequency of B chromosomes in the population studied, and several mechanisms may be involved, including selection, random transmission, and historical factors.

Among Coleoptera species, the studies reporting the presence of B chromosomes have generally focused on the presence or absence of this element and have not considered their frequency in the population or their molecular content [18,21,23,36]. The presence of B chromosomes was reported in representatives of the Cetoniinae and Scarabaeinae, subfamilies of Scarabaeidae [21,22]. The evolution of the Scarabaeinae karyotype appears to have occurred under diverse mechanisms of chromosomal rearrangements [37], which could have contributed to the origin of the B chromosome in this group.

### Molecular cytogenetic mapping of *C. cyanescens*

The hybridization of the *C*_*0*_*t*-1 DNA to the pericentromeric regions extending up to the long arms of *C. cyanescens* chromosomes is in agreement with the heterochromatin distribution pattern observed in this species [26]. Although heterochomatin analyses were not conducted in the present work, the accumulation of repeated DNAs in the pericentromeric region of the B suggests also the compartimentalization of heterochromatin in the same region. The formation of the heterochromatic chromocenters in the Phanaeini species [38,39] indicates that this mechanism of heterochromatin amplification may be involved in the formation of diphasic chromosomes, including the large pericentromeric block of the B chromosome.

The distribution of *C*_*0*_*t*-1 DNA in the A complement and the B chromosome suggests an intraspecific origin of the extra element and the occurrence of homogenization mechanisms in the heterochromatic regions between the B and A elements. Generally, B chromosomes of more recent origin are enriched in repetitive DNA sequences when compared with the genome from which they originated [1,23]. This enrichment is indicative of a massive amplification of repetitive sequences over a relatively short time-scale; and, it has also been suggested that repetitive sequences amplification may be a mechanism through which a chromosome fragment (as a neo-B chromosome) may become stabilized and selected [1,23]. This does not appear to be the case for *C. cyanescens*, indicating that the B chromosome may not have been recently established in this species. Although the data obtained indicates an intraspecific origin of the B chromosome, it was not possible to identify which chromosomal A element was involved in the process. However, the chromosomes carrying the 5S and 18S RNA genes are probably not involved in this process, as the B element does not contain rRNA gene sequences.

The cytogenetic mapping of the *LOA-like* non-LTR retrotransposon mostly to the pericentromeric regions, including those of the B chromosome, indicates the exchange of genetic material between the A and B chromosomes, implying that the B chromosome has coexisted with the A chromosomes during the period of transposition. However, it is not possible to reject the hypothesis that the B chromosome originated from a segment without *LOA-like* that was received later, by transposition. According to a previous report [40], B chromosomes can accumulate DNA from various sources, including transposable elements, and may affect the structure of the genome by ectopic recombination. A study in *Drosophila melanogaster* identified 25 transposon-mediated rearrangements by ectopic recombination in the region flanking the white locus [41]. The B chromosomes could act as a refuge for TEs, which in turn would generate structural variability in the whole genome. The hybridization that occurred in homologous regions, such as the pericentromeric regions, is another indication of recombination between the A complement and the B chromosome, and this recombination event could be explained by the chromocenter formation during the beginning of meiosis [37].

The present study is the first report on the cytogenetic mapping of a transposable element in coleopteran chromosomes. The *LOA* non-LTR retrotransposon was first isolated from the genome of *Drosophila silvestris*, a species that is endemic to the Hawaiian Islands [42]. These elements belong to evolutionarily younger clades of non-LTR retrotransposons [43], contain very few known elements, and have mostly been identified in *Drosophila*, *Aedes* and *Ciona* genomes [44].

The distribution of *LOA-like* elements in the chromosomes reinforces an evolutionary relationship between the A complement and the B chromosome at least in the pericentromeric area. Recent work involving the centromere-enriched retrotransposons indicates that these elements preferentially insert into the centromeric regions [45]. The *LOA-like* elements may have been maintained in the genome of *C. cyanescens* due to a possible functional role they play in the maintenance of the pericentromeric regions. The absence of *LOA-like* elements in the sex chromosomes suggests that sex differentiation occurs before the distribution of this transposable element into the genome. Subsequently, the suppression of recombination could have produced the differences observed in the distribution of TEs between the A complement and the sex chromosomes. These results suggest that *LOA-like* element could have been involved in the maintenance of the pericentromeric regions and might contribute to the origin of the B chromosome.

## Conclusions

The results obtained by the hybridization of *C*_*0*_*t*-1 DNA and the *LOA-like* non-LTR retrotransposon indicate that the origin of the B chromosome is associated with autosomal elements. The present study is the first report on the cytogenetic mapping of a transposable element in coleopteran chromosomes. Our work further suggests that TEs could also have been involved in the origin and evolution of the B chromosome in *C. cyanescens*.

## Methods

### Animal sampling and cytogenetic analysis

Fifty adult specimens of *Coprophanaeus cyanescens* (Olivier, 1789) (Coleoptera: Scarabaeidae: Scarabaeinae: Phanaeini) were obtained from Parque João Vasconcelos Sobrinho, Caruaru, Pernambuco State, Brazil. The specimens were collected in the wild according to Brazilian laws for environmental protection (wild collection permit, MMA/IBAMA/SISBIO n^o^. 2376–1). The experimental research on animals was conducted according to the international guidelines followed by São Paulo State University (Protocol no. 35/08 – CEEA/IBB/UNESP).

The testes were fixed in Carnoy solution (3:1 ethanol: acetic acid) and later stored at −20°C. The chromosome preparations were obtained by using the classical testicular follicle squashing technique. Conventional chromosome analysis was performed after staining the slides with 5% Giemsa.

### Chromosomal probe isolation

The DNA samples were obtained from frozen tissues collected from specimens. The procedure for extraction of genomic DNA followed the protocol previously described [46] with minor modifications. The quality and quantity of purified DNA was evaluated in 0.8% agarose gel and spectrophotometry.

Three sets of DNA sequences were used as probes for fluorescence *in situ* hybridization (FISH) as follow: (i) sequences for the 18S and 5S rRNA genes were obtained from cloned sequences of the dung beetle, *Dichotomius semisquamosus*[22]; (ii) sequences of the *LOA-like* non-LTR retrotransposon were obtained from *C. cyanescens* by PCR with the RF-Co (5’ CGC CTA CTT CAG GAC CAG AG 3’) and RR-Co (5’ AGA CTG CAG GCC GTA GAA AA 3’) primers [47]; (iii) *C*_*0*_*t*-1 DNA sequences were isolated from *C. cyanescens* based on the DNA re-association kinetics [48] with modifications [49].

PCR products from the non-LTR retrotransposons were inserted into the pGEM-T plasmid (Promega) according to the manufacturer’s recommendations, and the recombinant plasmids were used to transform competent *Escherichia coli* cells (Invitrogen, San Diego, CA, USA). The presence of the inserts in the recombinant plasmids was analyzed by PCR, and the recombinant clones were stored at −80°C. The recombinant plasmids were subjected to nucleotide sequencing using an Applied Biosystems sequencer (3500 Genetic Analyzer).

### Analysis of transposable elements

The *LOA-like* non-LTR retrotransposon sequences isolated by PCR from *C. cyanescens* were used as queries to detect related TEs in other genomes available from the Repbase ( http://www.girinst.org/repbase/) and NCBI (National Center for Biotechnology Information - http://www.ncbi.nlm.nih.gov/) databases. The search included whole genome shotgun contigs, nucleotide collections, and high throughput genomic sequences. Analysis of the recovered DNA sequences were performed with the LIRMM software (Laboratoire Le d’Informatique, Robotique et de Microélectronique of Montpellier) available online ( http://www.phylogeny.fr/) [50-52].

### Fluorescence *in situ* hybridization

The DNA probes were labeled by nick translation with biotin-11-dATP (Invitrogen) or digoxigenin-11-dUTP (Roche, Mannheim, Germany) by PCR. The FISH technique was performed according to a protocol adapted for Coleoptera [22]. The chromosome spreads were counterstained with DAPI (4', 6-diamidino-2-phenylindole), and the slides were mounted in Vectashield mounting medium (Vector, Burlingame, CA, USA). The images were captured using an Olympus DP71 digital camera coupled to a BX61 Olympus microscope and were optimized for brightness and contrast using Adobe Photoshop CS2 and Corel Photo-Paint 13.

## Abbreviations

CEEA: Comissão de Ética em Experimentação Animal; DAPI: 4' 6-Diamidino-2-Phenylindole; FISH: Fluorescence *In Situ* Hybridization; IBAMA: Instituto Brasileiro do Meio Ambiente e dos Recursos Naturais Renováveis; IBB: Instituto de Biociências de Botucatu; LIRMM: Laboratoire Le d’Informatique, Robotiqueet de Microélectronique of Montpellier; LTR: Long Terminal Repeat; MMA: Ministério do Meio Ambiente; NCBI: National Center for Biotechnology Information; PCR: Polymerase Chain Reaction; rDNA: ribosomal DNA; rRNA: ribosomal RNA; SISBIO: Sistema de Autorização e Informação em Biodiversidade; UNESP: Universidade Estadual Paulista; TE(s): Transposable Element(s).

## Competing interests

The authors declare that they have no competing interests.

## Authors’ contributions

SGO, RCM and CM contributed to the development of the hypothesis, specimen collection and preparation, and analysis and interpretation of data. SGO and CM drafted the first version of the manuscript. RCM revised the manuscript. All authors read and approved the final manuscript.

## Supplementary Material

Additional file 1 **Alignment of the *LOA *non-LTR retrotransposon nucleotide sequences from *Nasonia vitripennis *(Baggins-1_NVi) and *Coprophanaeus cyanescens *(Cc-1 to Cc-3).** The asterisks (*) indicate similarity in sequence, and the dashes (−) indicate indels. Click here for file
