# Supplementary material for: B chromosome in the beetle Coprophanaeus cyanescens (Scarabaeidae): emphasis in the organization of repetitive DNA sequences
Source: BMC Genet. 2012 Nov 6;13:96. doi: 10.1186/1471-2156-13-96 (PMC3506448; doi:10.1186/1471-2156-13-96)
Supplement: Additional file 1 — Alignment of the LOA non-LTR retrotransposon nucleotide sequences from Nasonia vitripennis (Baggins-1_NVi) and Coprophanaeus cyanescens (Cc-1 to Cc-3). The asterisks (*) indicate similarity in sequence, and the dashes (−) indicate indels. [file 1471-2156-13-96-S1.pdf]

Additional file 1: Alignment of the *LOA* non-LTR retrotransposon nucleotide sequences from *Nasonia vitripennis* (Baggins-1_NVi) and *Coprophanaeus cyanescens* (Cc-1 to Cc-3). The asterisks (*) indicate similarity in sequence, and the dashes (-) indicate indels.

```
Baggins-1_NVi     TGCTGCAGCAGGGCATGGACGTATTAGTCCCAGCCTTGGAGAAATTGTACCGAGCCTGTC
Cc-1              TGCTACAATAGGGCATTGATCTATCATCTCCACTTCTCTGCATTATATACATGGCCAGTT
Cc-2              TGCTACAATAGGGCATTGATCTATCATCTCCACTTCTCTGCATTATATACATGGCCAGTT
Cc-3              TGCTACAATAGGGCATTGATCTATCATCTCCACTTCTCTGCATTATATACATGGCCAGTT
                  **** **  ******* **  *** *   ***    *    *   * ***   *** **

Baggins-1_NVi     TGGCACTAGGATATGTGCCGGAAGAATGGGGGCAGGCGAGGGTGGCTTTCCTGCCCAAAC
Cc-1              TGGCATTGGTATATATGTCTGAAAAGTGGATGGAAACTACGGTGGTTTTTATATCCAAAC
Cc-2              TGGCATTGGTATATATGTCTGAAGAGTGGATGGAAACTACGGTGGTTTTTATATCCAAAC
Cc-3              TGGCATTGGTATATATGTCTGAAAAGTGGATGGAAACTACGGTGGTTTTTATATCCAAAC
                  ***** * * **** ** * *** * ***  * *  * * ***** ***  *  ******

Baggins-1_NVi     CAGGTAAGAC-----ACAACACGCGGTCGCAAAGGACTTCAGGCCAATCAGCATGACCTC
Cc-1              TGGGCCCTACTTCTTACAACTGGC-----CAAAAGCATTCTGGCTAATCAGTCTGACGTC
Cc-2              TGGGCCCTACTTCTTACAACTGGC-----CAAAAGCATTCTGGCTAATCAGTCTGACGTC
Cc-3              TGGGCCCTGCTTCTTACAACTGGC-----CAAAAGCATTCTGGCTAATCAGTCTGACGTC
                    **     *     *****  **     **** *  *** *** ******  **** **

Baggins-1_NVi     GTTTTTACTCAAAACCCTGGAAAGGCTGGTTGACAGATATATCGAGGA
Cc-1              TTTCCTGCTAAAAGCCATGAAAAGGTTGATTGAGCGACGTATTAGGGA
Cc-2              TTTCCTGCTAAAAGCCATGAAAAGGTTGATTGAGCGACGTATTAGGGA
Cc-3              TTTCCTGCTAAAAGCCATGAAAAGGTTGATTGAGCGACGTATTAGGGA
                   **  * ** *** ** ** ***** ** ****  **  ***   ***
```
